# Supplementary material for: A whole-ecosystem experiment reveals flow-induced shifts in a stream community
Source: Commun Biol. 2022 May 5;5:420. doi: 10.1038/s42003-022-03345-5 (PMC9072309; doi:10.1038/s42003-022-03345-5)
Supplement: Supplementary file 2 — Supplementary Information [file 42003_2022_3345_MOESM2_ESM.pdf]

Supplementary Information for:

A whole-ecosystem experiment reveals flow-induced shifts in a stream community

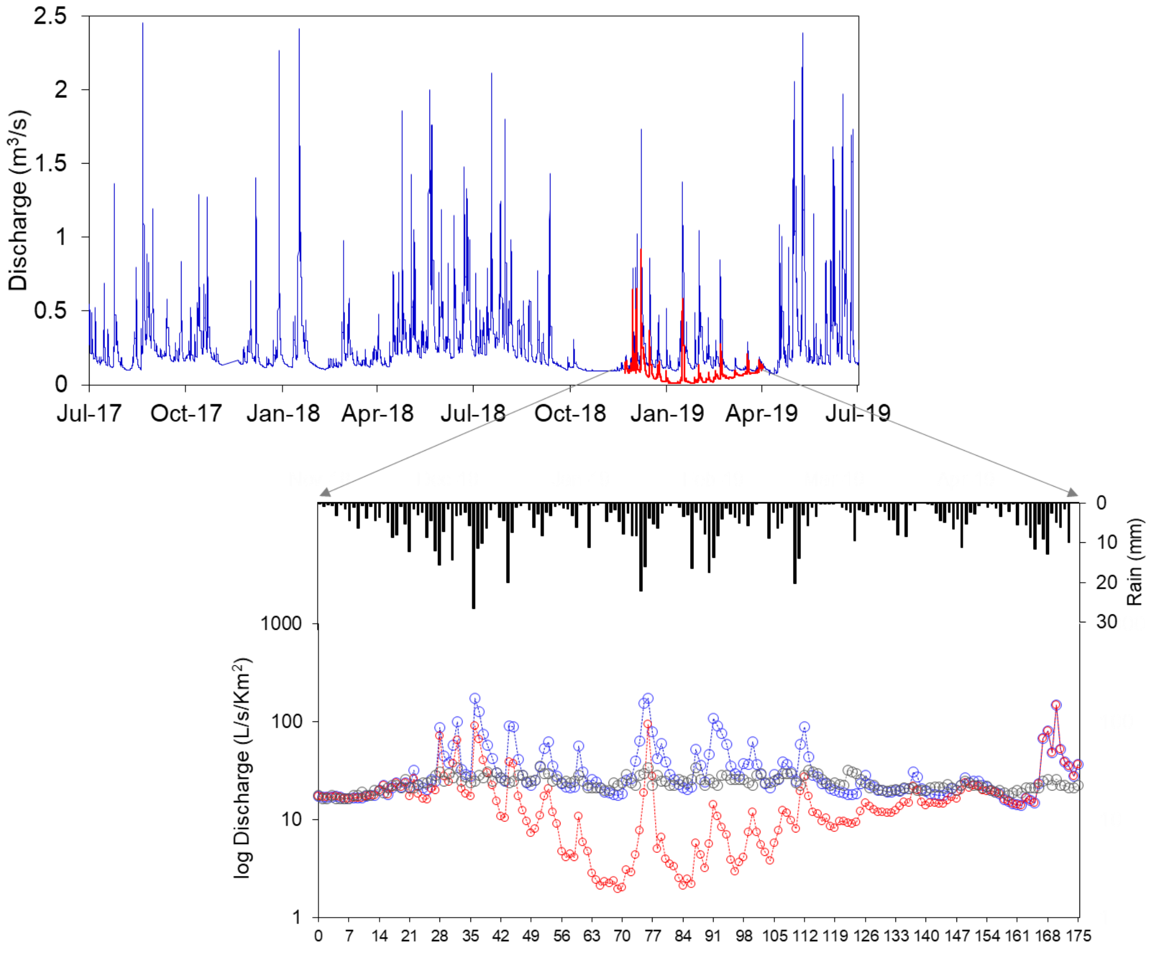

Supplementary Figure 1. Chalpi Norte stream hydrograph from 10-min water levels converted to discharge for the upstream site (blue line) and manipulated downstream site (red line). A close-up to flow manipulation experiment show daily averaged precipitation in the top (bars) and drainage-area normalized discharge for the upstream (blue open circles) and downstream site (red open circles) from the diversion in the Chalpi Norte stream, and the reference stream (gray open circles).

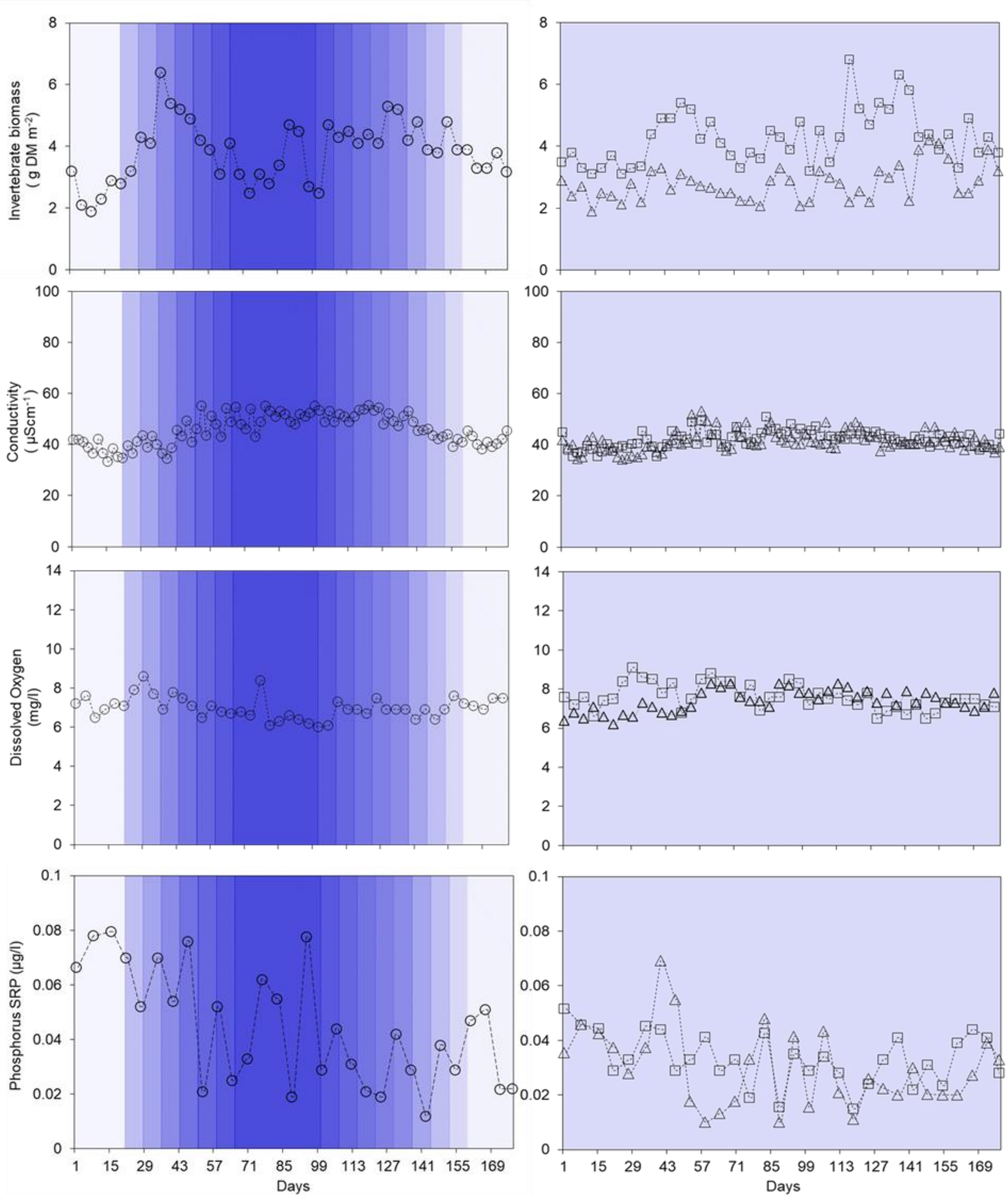

Supplementary Figure 2. Invertebrates' biomass **(a),** conductivity **(b),** dissolved oxygen **(c),** and phosphorus **(d)** measured during flow manipulation on the experimental site (circles, blue shades, see Figure 2a), and reference conditions (gray shade) at the upstream reach of the experimental site (squares) and the upstream reach of the control site (triangles), the downstream reach of the control site shows similar response as the upstream reach, see Table 1.

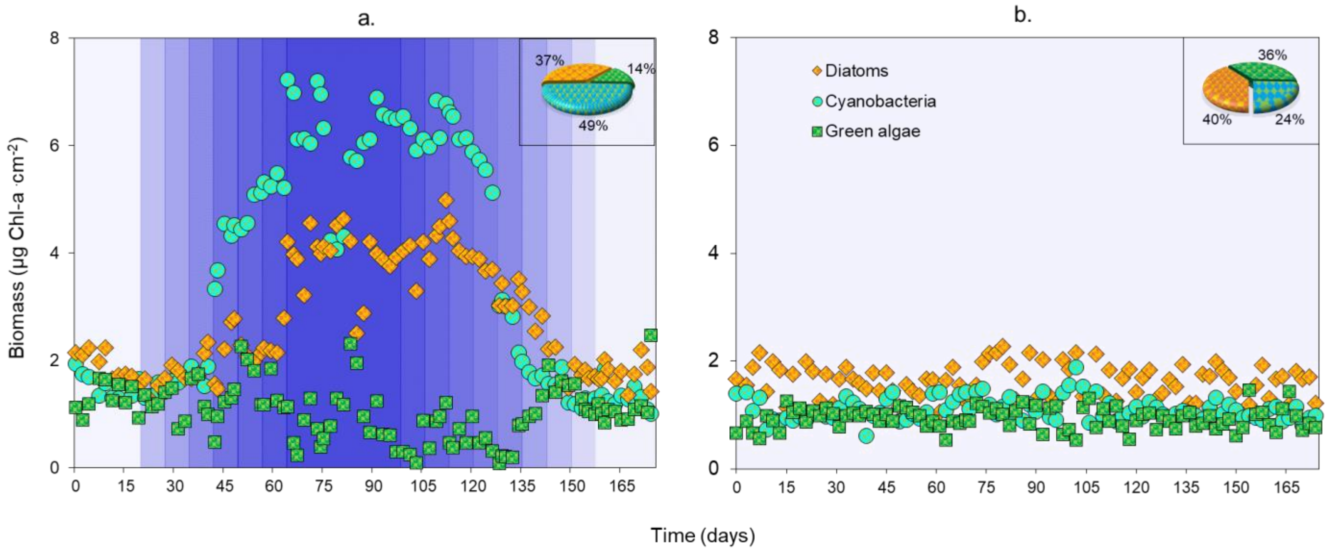

Supplementary Figure 3. Benthic cyanobacteria (turquois circles), diatoms (orange rhomboids), and green algae (green squares) biomass temporal measurements (days) on the manipulated stream (a) (flow reductions and flow recovery-blue shades, see Figure 3a) and the control stream (b) (upstream and the downstream sites show a similar response, see Table 1), right-top frames show of color-coded groups proportion (%).

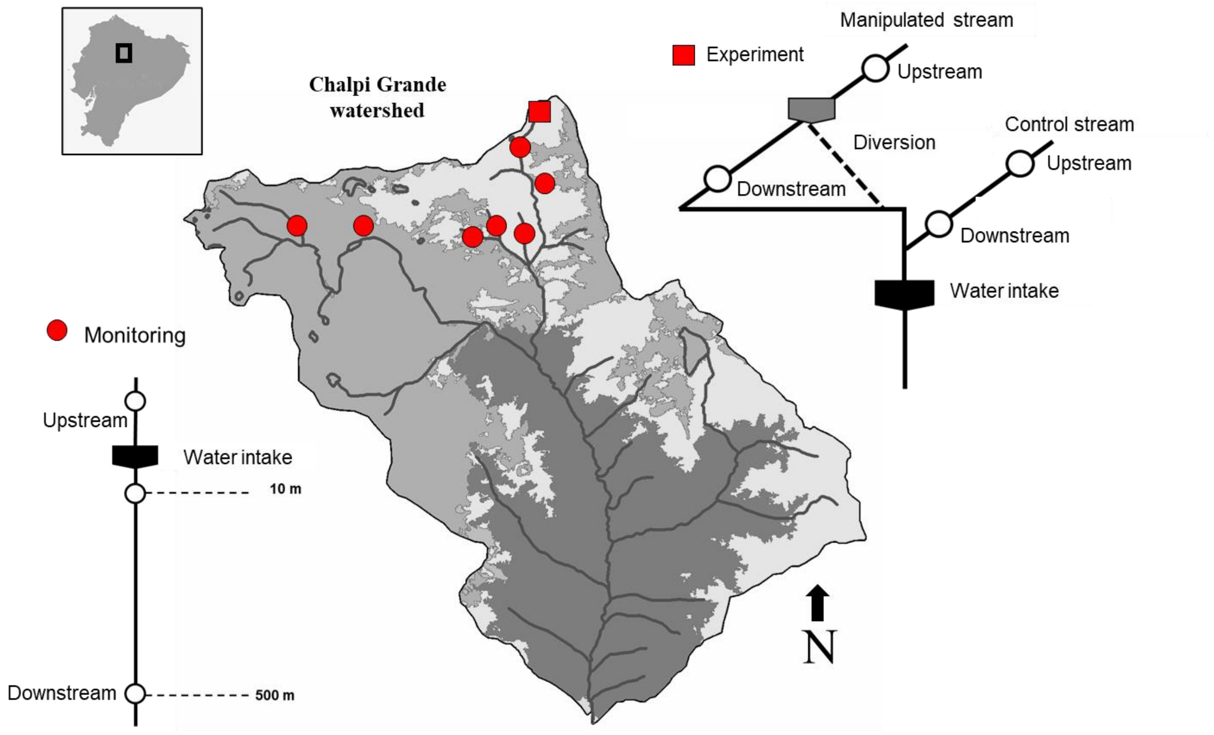

Supplementary Figure 4. Ecuadorian study sites in the headwaters of the Chalpi Grande watershed. The experimental site (red square) in the Chalpi Norte stream was located above the water intake from the supply system (black connector). The BACI design comprised of an upstream/free-flowing site and a downstream/manipulated site (white circles) in the manipulated stream, and an upstream and downstream site (white circles) in the control stream used as a reference. Nearby streams from the water supply system (red circles) monitored upstream water intakes and downstream at different distances (10 m and 500 m) (white circles).

Supplementary Table 1. Study sites where benthic cyanobacteria responded to flow reduction (n = 53), quantitative data used (n =33) to calculate the percent of flow remaining in streams and a factor of benthic cyanobacteria increase or decrease according to reported baseline flow conditions (either temporal or spatial).

Freshwater system	Landscape	Elevation (m) a.s.l.	Location / Country	Discharge (m^3/s)	Cyanobacteria dominant taxa	Response to flow reduction	Reference
Murray-Darling	Floodplain	3.6	Australia	11.57	*Lyngbya*	Increase	Burns and Walker 2000
Colorado river	Great basin desert	944	United States	414	*Oscilatoria*	Increase	Benenati et al. 2000
Mataura *	Floodplain	12	New Zealand	60	*Phormidium*	Increase	Hamill 2001
Waikanae*	Floodplain	9	New Zealand	28	*Oscillatoria*	Increase	Hamill 2001
Muga*	Coastal mountains	310	Spain	109.7	*Phormidium*	Decrease	Aboal et al. 2002
Parrizal*	Coastal mountains	650	Spain	107.5	*Phormidium*	Decrease	Aboal et al. 2002
Okuku	Grassland	654	New Zealand	4.64	*Phormidium*	Increase	Suren et al. 2003
Waipara	Grassland	105	New Zealand	3.06	*Phormidium*	Increase	Suren et al. 2003
Llobregat	Mediterranean	N/A	Spain	0.25	*Oscilatoria*	Increase	Sabater et al. 2003
Llobregat	Mediterranean	N/A	Spain	0.17	*Oscillatoria*	Increase	Vilalta et al. 2004
Alharabe	Forested highlands	1000	Spain	42.5	*Mycrosystis*	Increase	Aboal et al. 2005
Alharabe*	Forested highlands	1000	Spain	42.5	*Rivularia*	Decrease	Aboal et al. 2005
Motueka*	Coastal mountains	182	New Zealand	23.3	*Oscillatoria*	Increase	Wood et al. 2006
Waikanae*	Coastal mountains	101	New Zealand	19.4	*Oscillatoria*	Increase	Wood et al. 2006
Motupiko*	Coastal mountains	100	New Zealand	11.4	*Oscillatoria*	Increase	Wood et al. 2006
Tikotu*	Coastal mountains	78	New Zealand	21.5	*Oscillatoria*	Increase	Wood et al. 2006
Waikato*	Coastal mountains	90	New Zealand	15.7	*Phormidium*	Increase	Wood et al. 2006
Utakura*	Coastal mountains	56	New Zealand	9.44	*Mycrosystis*	Increase	Wood et al. 2006
Yabba*	Floodplain	65.4	Australia	N/A	*Lyngbya*	Increase	Seifert et al. 2007
Brisbane*	Floodplain	213	Australia	N/A	*Lyngbya*	Increase	Seifert et al. 2007
Providence*	Headwaters	1800	United States	1.82	*Anabaena*	Increase	Brown et al. 2008
Duff*	Headwaters	1500	United States	0.88	*Anabaena*	Increase	Brown et al. 2008
Bull*	Headwaters	2160	United States	8.58	*Anabaena*	Increase	Brown et al. 2008
Teakettle*	Headwaters	2005	United States	34.41	*Anabaena*	Increase	Brown et al. 2008
Fuirosos*	Mediterranean	700	Spain	0.025	*Phormidium*	Increase	Tornés & Sabater 2010
Wainuiomata	Floodplain	0	New Zealand	0.95	*Phormidium*	Increase	Heath et al. 2011
Mangaroa	Floodplain	220	New Zealand	3.18	*Phormidium*	Increase	Heath et al. 2011
Hutt in Boulcott	Floodplain	0	New Zealand	20.4	*Phormidium*	Increase	Heath et al. 2011
Hutt in Silverstream	Floodplain	0	New Zealand	26.14	*Phormidium*	Increase	Heath et al. 2011
Hutt in Whakatikei	Floodplain	122	New Zealand	14.15	*Phormidium*	Increase	Heath et al. 2011
Hutt in Akatarwa	Floodplain	145	New Zealand	10.22	*Phormidium*	Increase	Heath et al. 2011
Hutt in Te Maura	Floodplain	140	New Zealand	11.14	*Phormidium*	Increase	Heath et al. 2011
Hutt	Floodplain	232	New Zealand	15.21	*Phormidium*	Increase	Heath et al. 2013
Li	Headwaters	740	Norway	3.52	*Phormidium*	Increase	Schneider et al. 2015
Eel	Coastal mountains	0	United States	260	*Anabaena*	Increase	Bouma-Gregson et al. 2017
Makakahi	Pastoral land use	22	New Zealand	3.18	*Phormidium*	Increase	Wood et al. 2017
Manawatu	Pastoral land use	45	New Zealand	73.4	*Phormidium*	Increase	Wood et al. 2017
Mangatainoka	Native vegetation	24	New Zealand	2.13	*Phormidium*	Increase	Wood et al. 2017
Oroua	Pastoral land use	32	New Zealand	7.1	*Phormidium*	Increase	Wood et al. 2017
Orouakeretaki	Pastoral land use	79	New Zealand	1.42	*Phormidium*	Increase	Wood et al. 2017
Tiraumea	Pastoral land use	80	New Zealand	7.21	*Phormidium*	Increase	Wood et al. 2017
Tokomaru	Native vegetation	100	New Zealand	1.25	*Phormidium*	Increase	Wood et al. 2017
Nederbach*	Headwaters	980	Austria	N/A	*Chamaesiphon*	Increase	Aigner et al. 2018
Isar*	Headwaters	980	Austria	2.2	*Syneschococcales*	Increase	Aigner et al. 2018
Waipara	Intensive agriculture	194	New Zealand	0.9	*Phormidium*	Increase	McAllister et al. 2018
Ashley	Intensive agriculture	1802	New Zealand	10.2	*Phormidium*	Increase	McAllister et al. 2018
Selwyn	Intensive agriculture	256	New Zealand	2	*Phormidium*	Increase	McAllister et al. 2018
Orari	Intensive agriculture	171	New Zealand	6.2	*Phormidium*	Increase	McAllister et al. 2018
Temuka	Intensive agriculture	23	New Zealand	3.4	*Phormidium*	Increase	McAllister et al. 2018
Opihi	Intensive agriculture	185	New Zealand	8.7	*Phormidium*	Increase	McAllister et al. 2018
Te ana a wai	Intensive agriculture	112	New Zealand	1.8	*Phormidium*	Increase	McAllister et al. 2018
Pareora	Intensive agriculture	138	New Zealand	1.4	*Phormidium*	Increase	McAllister et al. 2018
Ter	Mid-section	1100	Spain	0.01	*Oscillatoria*	Increase	Espinosa et al. 2020

* Qualitative observations

Supplementary References

1. K. Bouma-Gregson, R.M. Kudela, M.E. Power. Widespread anatoxin-a detection in benthic cyanobacterial mats throughout a river network. *PLoS One* 13(5): e0197669. (2018).
2. E.P. Benenati, J.P. Shannon, D.W. Blinn, K.P. Wilson, S.J. Hueftle. Reservoir–river linkages: Lake Powell and the Colorado River, Arizona. *Journal of the North American Benthol. Soc.* 19(4): 742-755. (2000).
3. T.G. McAllister, S.A. Wood, J. Atalah, I. Hawes. Spatiotemporal dynamics of Phormidium cover and anatoxin concentrations in eight New Zealand rivers with contrasting nutrient and flow regimes. *Science of the Total Environment* 612: 71-80. (2018).
4. M.W. Heath, S.A. Wood, K.G. Ryan. Spatial and temporal variability in Phormidium mats and associated anatoxin-a and homoanatoxin-a in two New Zealand rivers. *Aquatic Microbial Ecology*, 64(1), 69-79. (2011).
5. M.W. Heath, S.A. Wood, K. A. Brasell, R. G. Young, K. G. Ryan. Development of habitat suitability criteria and in-stream habitat assessment for the benthic cyanobacteria Phormidium. *River Research and Applications* 31(1): 98-108. (2013).
6. M. Seifert, G. McGregor, G.Eaglesham, W. Wickramasinghe, G. Shaw. First evidence for the production of cylindrospermopsin and deoxy-cylindrospermopsin by the freshwater benthic cyanobacterium, Lyngbya wollei (Farlow ex Gomont) Speziale and Dyck. *Harmful algae* 6: 73-80. (2007).
7. M. Aboal, M.A. Puig, P. Mateo, E. Perona. Implications of cyanophyte toxicity on biological monitoring of calcareous streams in north-east Spain. *Journal of Applied Phycology* 14(1): 49-56. (2002).
8. M. Aboal, M. A. Puig, A. D. Asencio. Production of microcystins in calcareous Mediterranean streams: the Alharabe River, Segura River basin in south-east Spain. *Journal of Applied Phycology* 17(3): 231-243. (2005).
9. K.D. Hamill. Toxicity in benthic freshwater cyanobacteria (blue-green algae): First observations in New Zealand. 35(5): 1057-1059. (2001).
10. A. M. Suren, B.J.F. Biggs, C. Kilroy, L. Bergey. Benthic community dynamics during summer low-flows in two rivers of contrasting enrichment 1. Periphyton. *New Zealand Journal of Marine and Freshwater Research*. 37(1): 53-70. (2003).
11. A. Burns, K. F. Walker. Effects of water level regulation on algal biofilms in the River Murray, South Australia. *Regulated Rivers: Research & Management: An International Journal Devoted to River Research and Management.* 16(5): 433-444. (2000).
12. A. Abonyi, É. Ács, A. Hidas, I. Grigorszky, G. Várbíró, G. Borics, K.T. Kiss. Functional diversity of phytoplankton highlights long-term gradual regime shift in the middle section of the Danube River due to global warming, human impacts and oligotrophication. *Freshwater Biology*. 63(5): 456-472. (2018).
13. E. Vilalta, H. Guasch, I. Muñoz, A. Romani, F. Valero, J. J. Rodriguez, R. Alcaraz, S. Sabater. Nuisance odours produced by benthic cyanobacteria in a Mediterranean river. *Water Science and Technology* 49(9): 25-31. (2004).
14.

15. S. C. Schneider. Greener rivers in a changing climate? - Effects of climate and hydrological regime on benthic algal assemblages in pristine streams. *Limnologica*. 55: 21-32. (2015).
16. S. Aigner, K. Herburger, A. Holzinger, U. Karsten. Epilithic Chamaesiphon (Synechococcales, Cyanobacteria) species in mountain streams of the Alps—interspecific differences in photo-physiological traits. *Journal of applied phycology*. 30(2): 1125-1134. (2018).
