# Supplementary material for: A whole-ecosystem experiment reveals flow-induced shifts in a stream community
Source: Commun Biol. 2022 May 5;5:420. doi: 10.1038/s42003-022-03345-5 (PMC9072309; doi:10.1038/s42003-022-03345-5)
Supplement: Supplementary file 3 — Reporting Summary [file 42003_2022_3345_MOESM3_ESM.pdf]

nature research

Corresponding author(s): COMMSBIO-21-0124C

Last updated by author(s): 2022/02/21

Reporting Summary

Nature Research wishes to improve the reproducibility of the work that we publish. This form provides structure for consistency and transparency in reporting. For further information on Nature Research policies, see our Editorial Policies and the Editorial Policy Checklist.

Statistics

For all statistical analyses, confirm that the following items are present in the figure legend, table legend, main text, or Methods section.

n/a	Confirmed	
☐	☒	The exact sample size (*n*) for each experimental group/condition, given as a discrete number and unit of measurement
☐	☒	A statement on whether measurements were taken from distinct samples or whether the same sample was measured repeatedly
☐	☒	The statistical test(s) used AND whether they are one- or two-sided<br>*Only common tests should be described solely by name; describe more complex techniques in the Methods section.*
☐	☒	A description of all covariates tested
☐	☒	A description of any assumptions or corrections, such as tests of normality and adjustment for multiple comparisons
☐	☒	A full description of the statistical parameters including central tendency (e.g. means) or other basic estimates (e.g. regression coefficient) AND variation (e.g. standard deviation) or associated estimates of uncertainty (e.g. confidence intervals)
☒	☐	For null hypothesis testing, the test statistic (e.g. *F*, *t*, *r*) with confidence intervals, effect sizes, degrees of freedom and *P* value noted<br>*Give P values as exact values whenever suitable.*
☒	☐	For Bayesian analysis, information on the choice of priors and Markov chain Monte Carlo settings
☒	☐	For hierarchical and complex designs, identification of the appropriate level for tests and full reporting of outcomes
☒	☐	Estimates of effect sizes (e.g. Cohen's *d*, Pearson's *r*), indicating how they were calculated

Our web collection on statistics for biologists contains articles on many of the points above.

Software and code

Policy information about availability of computer code

Data collection	The datasets generated during and/or analysed during the current study are available from the corresponding author on reasonable request.
Data analysis	All the analyses performed during the current study used open source software and the code generated is available from the corresponding author on reasonable request.

For manuscripts utilizing custom algorithms or software that are central to the research but not yet described in published literature, software must be made available to editors and reviewers. We strongly encourage code deposition in a community repository (e.g. GitHub). See the Nature Research guidelines for submitting code & software for further information.

Data

Policy information about availability of data

All manuscripts must include a data availability statement. This statement should provide the following information, where applicable:

- Accession codes, unique identifiers, or web links for publicly available datasets
- A list of figures that have associated raw data
- A description of any restrictions on data availability

The datasets generated during and/or analysed during the current study are available from the corresponding author on reasonable request.

Field-specific reporting

Please select the one below that is the best fit for your research. If you are not sure, read the appropriate sections before making your selection.

☐ Life sciences ☐ Behavioural & social sciences ☒ Ecological, evolutionary & environmental sciences

For a reference copy of the document with all sections, see nature.com/documents/nr-reporting-summary-flat.pdf

Ecological, evolutionary & environmental sciences study design

All studies must disclose on these points even when the disclosure is negative.

Study description	To determine cyanobacteria regime shifts in a potable water supply system in the tropical Andes, we conducted a whole ecosystem-scale experiment in which we systematically diverted 20 to 90% of streamflow and measured resulting ecological responses. Cyanobacteria dramatically increased with a 60% flow reduction and this tipping point was related to water temperature and nitrate concentration increases, both known to boost algal productivity. We supplemented our experiment with a regional survey collecting > 1450 flow-algal measurements at streams varying in water abstraction levels. We confirmed the tipping point flow value, albeit at a slightly lower threshold (40-50%). The experimental flow reduction favored cyanobacteria dominance over other types of algae that also changed (i.e., diatoms increased, and green algae remained stable) but the proportion of the three types returned to baseline conditions after flow was reinstated
Research sample	We addressed regime shifts in CyanoHABs and other benthic components (e.g., diatoms, green algae, and grazing invertebrates) using a whole ecosystem experiment in a mountain river system of the tropical Andes.
Sampling strategy	On the experimental and control sites we measured biotic, physical, and chemical in situ parameters every two days (n = 1760), and nutrients and invertebrates every seven days (n = 500) for the duration of the flow manipulation (~0.5 years). On the monitored sites, we measured biotic, physical, and chemical in situ parameters every seven days (n = 1456) and nutrients and invertebrates every 30 days (n = 336).
Data collection	To measure Chl-a from cyanobacteria and benthic algae on artificial substrates, we used a BenthoTorch® (bbe Moldaenke GmbH, Germany) on unglazed ceramic plates (200 mm x 400 mm) with a grid of 25 squares of 2500 mm2 to allow algal accrual on a standardized surface. We allowed 21 days for colonization (based on previous observations) and then we placed all substrates (5) at the beginning of the experiment. We performed five readings on five squares randomly selected within each plate. To consider the effect of benthic invertebrates to flow variations, we sampled stream sites using a Surber net (mesh size = 250 µm, area = 0.0625 m2).
Timing and spatial scale	We conducted our experimental flow manipulation between October 2018 and April 2019 in a mainly rain-fed stream (29). The experiment manipulated natural flows encompassing stable low flows and sporadic spates characterizing the high temporal variability of headwaters. We set up a full Before-After/Control- Impact (BACI) experiment to evaluate ecosystem variables under natural and manipulated flow conditions. The experimental site was comprised of an upstream/free-flowing reach (L = 25 m) (reference conditions), located ~32 m above the ecohWe started diversions to maintain in the meander 100, 80, 60, 50, 40, 30, and 20% of the incoming flow for 7-day periods (based on local observations of benthic algal colonization); then we maintained 10% of the upstream flow for 36 days. We started to return flow gradually to recover 20, 30, 40, 50, 60, 80, and 100% of the upstream flow. ydraulic structure and a downstream/regulated reach (L = 97 m) located immediately below the flow manipulation structure. The control site was located in a free-flowing stream, a tributary of the Chalpi Norte stream, with an upstream reach separated from a downstream reach by a distance of 16 m.
Data exclusions	No data were excluded from the original design of the experiment and monitoring.
Reproducibility	Our experiment was designed to replicate in other high-altitude streams where water for human supply and irrigation are located.
Randomization	For biotic sampling, we ensured to measure cyanobacteria and benthic algae (diatoms and green algae) randomly by selecting quadrants within the unglazed ceramic plate. We assigned number codes and used open source code to identify the sample. For benthi invertebrates we sampled randomly within the reach, and for environmental variables we sampled continuously in the same location. The experiment and monitoring sampling was undertaken by the lead field researcher to reduce variability.
Blinding	The monitoring data from regional streams and data from the experimental stream provided the baseline information of the range of chlorophyll concentrations for high-altidude streams. We assessed conditions during different climatic conditions to cover temporal variations.

Did the study involve field work? ☒ Yes ☐ No

Field work, collection and transport

Field conditions	High-altitude streams in the Andean region of Ecuador originate in glacial and moorlands that have different levels of conservation governance. Our study was conducted in the Cayambe-Coca National Park where no other human activities besides water extraction are allowed.
Location	At 3800 m of altitude streams present low temperatures and high radiation, precipitation from the Amazon region presents a marked seasonality that contrast with the Pacific region, therefore these headwaters provide nearly 70% of the water to main city of Quito, in

	Ecuador. Streams at this elevation never freeze and have no forested areas to provide organic matter, the highest and natural trophic level are macronivertebrates and an introduced species of trout
Access & import/export	To set up the flow diversion structure we had to conduct to obtain a special permit to minimize any environmental impact, we also had to build and install the structure in 6 hours. We hired local people to conduct the work at this altitude and under low temperature conditions. To sample every two days, we installed our field station in the closest town and drove to the main city every week to bring sample to the laboratory.
Disturbance	During the project we experienced a 100-year rain event that overflowed our structure and damaged a first diversion structure installed prior the rainy season. We had to build a second structure during the dry season months and removed the structure prior the rainy season of the next year.

Reporting for specific materials, systems and methods

We require information from authors about some types of materials, experimental systems and methods used in many studies. Here, indicate whether each material, system or method listed is relevant to your study. If you are not sure if a list item applies to your research, read the appropriate section before selecting a response.

Materials & experimental systems

n/a	Involved in the study	
☒	☐	Antibodies
☒	☐	Eukaryotic cell lines
☒	☐	Palaeontology and archaeology
☐	☒	Animals and other organisms
☒	☐	Human research participants
☒	☐	Clinical data
☒	☐	Dual use research of concern

Methods

n/a	Involved in the study	
☒	☐	ChIP-seq
☒	☐	Flow cytometry
☒	☐	MRI-based neuroimaging

Animals and other organisms

Policy information about studies involving animals; ARRIVE guidelines recommended for reporting animal research

Laboratory animals	The study did not involve laboratory animals.
Wild animals	The study did not involve wild animals.
Field-collected samples	Field collected water samples were kept in coolers with ice packs and covered from direct light. In the laboratory, samples were processed within 4-hours for nutrients.
Ethics oversight	Cornell Soil and Water laboratory provided the ethics oversight of protocols and chain of custody of samples analyzed in the laboratory of the Water Supply Company in Ecuador.

Note that full information on the approval of the study protocol must also be provided in the manuscript.
